# Sex Differences in the Associations of Physical Activity and Planetary Health Diet with Obesity and Depressive Symptoms Among Adolescents in Zhejiang Province: An Observational Study

**DOI:** 10.3390/nu18081232

**Published:** 2026-04-14

**Authors:** Qu Lu, Manman Chen, Jiahui Wang, Yuankai Zhao, Zichen Ye, Jie Hu, Jia Meng, Juanjuan Li, Yu Shen, Fang Gu, Yu Jiang, Bin Dong

**Affiliations:** 1 School of Health Policy and Management, Chinese Academy of Medical Sciences and Peking Union Medical College, Beijing 100730, China; qulu167@163.com (Q.L.); anniewangjiahui@163.com (J.W.); ye18700579760@163.com (Z.Y.); 2 School of Population Medicine and Public Health, Chinese Academy of Medical Sciences and Peking Union Medical College, Beijing 100730, China; chenmm@pumc.edu.cn (M.C.); z4311652@163.com (Y.Z.); 3 School of Pharmacy and Medical Sciences, Griffith University, Nathan, Brisbane, QLD 4111, Australia; jie.hu@griffith.edu.au; 4 Faculty of Medicine, The University of Queensland, St Lucia, Brisbane, QLD 4072, Australia; 5 Division of School Health, Department of Nutrition and Food Safety, Zhejiang Provincial Center for Disease Control and Prevention, Hangzhou 310051, China; jmeng@cdc.zj.cn (J.M.); jjli@cdc.zj.cn (J.L.); yshen@cdc.zj.cn (Y.S.); 6 Institute of Child and Adolescent Health, School of Public Health, Peking University, Beijing 100871, China

**Keywords:** planetary health diet, physical activity, adolescent, co-occurrence

## Abstract

**Background**: Adolescent obesity and depressive symptoms have increased concurrently, often presenting as co-occurrence. However, evidence on the timing of physical activity (e.g., weekday vs. weekend) and adherence to planetary health diets remains limited. This study examined these associations among adolescents in Zhejiang Province from 2022 to 2024. **Methods**: A total of 261,495 adolescents aged 11–18 years were included. Physical activity (PA) and dietary behaviors were assessed through the China Common Disease and Risk Factor Surveillance among Students questionnaire (reliability: Cronbach’s α = 0.84, validity: RMSEA = 0.07). The plant-based Planetary Health Diet Index (PHDI-green) adherence was defined as consuming at least one daily serving of both vegetables and fruits. Depressive symptoms were measured using the Center for Epidemiologic Studies Depression (CES-D) scale, and co-occurrence was defined as the coexistence of obesity and depressive symptoms. Temporal trends were tested using χ^2^ tests. Sex-stratified logistic regression, restricted cubic spline analyses, and population attributable fraction (PAF) analyses were applied. **Results**: From 2022 to 2024, obesity (*p* for trend = 0.013) and depressive symptoms (*p* for trend = 0.003) increased significantly, while co-occurrence remained stable (*p* for trend = 0.058). Boys had higher obesity and co-occurrence, whereas girls showed higher depressive symptoms (all *p* < 0.001). Higher weekly PA, greater weekend PA and PHDI-green adherence were associated with reduced odds of obesity in both sexes (all *p* < 0.001). Weekend PA showed stronger associations with depressive symptoms among girls, while PHDI-green showed stronger inverse associations in boys (*p* for sex difference < 0.001). PAF analyses suggested that low weekend PA accounted for substantial proportions of cases (girls: obesity 10.17%, depressive symptoms 31.30%, co-occurrence 35.64%). Joint adherence to adequate PA and PHDI-green conferred the lowest odds of co-occurrence (boys: OR = 0.40, 95% CI: 0.34–0.46; girls: OR = 0.33, 95% CI: 0.26–0.43). **Conclusions**: Adherence to the Planetary Health Diet may be particularly relevant for boys, whereas PA—especially weekend PA—may be more strongly associated with health outcomes among girls. These findings suggest the importance of sex-specific and time-targeted behavioral strategies for obesity, depressive symptoms, and their co-occurrence in adolescents.

## 1. Introduction

Adolescent obesity and mental health problems have risen concurrently over recent decades, posing a major global public health challenge. From 1990 to 2021, the global prevalence of obesity among children and adolescents has nearly tripled and projections indicate that approximately 254 million children and adolescents may be living with obesity by 2030 if current trends persist [1,2]. A similar pattern has been observed in China. Among individuals aged 7–18 years, the prevalence of simple obesity rose dramatically from 0.03% to 5.03%, exceeding a 150-fold increase [3].

Meanwhile, global research has shown that depressive symptoms affect an estimated one in five adolescents worldwide, with prevalence continuing to increase across regions [4]. In China, depressive symptoms are reported in 26.17% of children and adolescents, with subclinical depressive symptoms accounting for as much as 24.5% [5]. Longitudinal studies have demonstrated that subclinical depressive symptoms are not benign; follow-up findings indicate that nearly two-thirds of affected adolescents later progress to clinically diagnosable depressive disorders [6] and may also be at increased risk of extreme outcomes such as self-harm and suicide [7].

Notably, obesity and depressive symptoms often coexist and interact through mechanisms such as physiological inflammation, neuroendocrine dysregulation and psychosocial stress [8], contributing to the phenomenon of co-occurrence, defined as the presence of two or more chronic conditions within an individual [9]. Among adolescents, this co-occurrence can exacerbate long-term health risks, including cardiovascular disease and impaired quality of life, and may amplify the burden of physical and mental health issues [10,11].

Physical activity (PA) and dietary behaviors are considered important modifiable factors influencing adolescent obesity, depressive symptoms and their co-occurrence [12,13,14]. Increasing attention has been directed toward the temporal and distributional characteristics of PA. Emerging concepts such as the “weekend warrior” highlight that the concentration of PA within specific time periods may confer health effects distinct from those associated with evenly distributed activity throughout the week [15]. The Planetary Health Diet Index (PHDI), derived from the EAT–Lancet framework for sustainable diets, emphasizes a plant-based dietary pattern that balances human health and environmental sustainability [16]. The plant-based Planetary Health Diet Index (PHDI-green) further characterizes the variety and consumption frequency of plant-origin foods, with particular emphasis on produce such as fruits and vegetables. Evidence from prior research indicates that greater consumption of these foods is linked to a lower likelihood of major depressive disorder [17].

Adolescent development is a critical period in which sex disparities are particularly pronounced. Boys are more inclined to engage in strength and endurance exercise, which is associated with higher testosterone levels [18]. In contrast, girls tend to place greater emphasis on dietary behaviors, potentially influenced by body image concerns [19]. These sex-specific behavioral patterns may lead to differential effects on obesity, depressive symptoms and their co-occurrence. Despite these insights, evidence integrating both overall volume and temporal distribution of PA, as well as adherence to PHDI-green, in relation to adolescent obesity, depressive symptoms and the comorbidity of these conditions remains limited. Moreover, potential sex-specific associations with these health outcomes have not been adequately explored.

Therefore, using data from the Zhejiang Province Surveillance of Common Diseases among Primary and Secondary School Students from 2022 to 2024, the present study investigated the prevalence and temporal patterns of obesity, depressive symptoms, and their co-occurrence in adolescents, and to examine sex-specific associations of PA characteristics and adherence to PHDI-green with these health outcomes. We hypothesized that higher PA levels, particularly temporally concentrated patterns such as the “weekend warrior” phenotype, together with stronger adherence to PHDI-green, would be associated with lower odds of obesity, depressive symptoms, and their co-occurrence among adolescents, with potential differences by sex. By explicitly addressing sex disparities, this study aims to provide evidence to support the design of more precise and sex-responsive PA and dietary interventions for improving adolescent health.

## 2. Materials and Methods

### 2.1. Study Design and Participants

This study is based on the China Common Disease and Risk Factor Surveillance among Students (CCDRFSS), a nationwide surveillance system conducted in China [20]. The survey collected demographic characteristics (e.g., date of birth, sex, grade level, and parental co-residence) as well as data on anthropometric measures and health conditions. A secondary analysis was conducted using routine health examination data of adolescents aged 11–18 years in Zhejiang Province between 2022 and 2024. Participants were excluded if they had (1) a history of severe drug allergy, (2) metal implants, or (3) major systemic diseases (such as cardiovascular, hepatic, or renal conditions), as well as any other contraindications to health examination.

The survey was conducted between 2022 and 2024 among middle school students in Zhejiang Province, China, covering both urban and rural areas across the province. To improve representativeness across regions and school categories, a stratified multistage cluster sampling strategy was applied. All prefecture-level administrative areas within the province were included, covering 11 cities and 90 districts/counties. In urban areas, five secondary schools were randomly chosen at each site, consisting of two junior high schools, two general senior high schools, and one vocational high school. In rural areas, three secondary schools were selected, including two junior high schools and one senior high school. Within each selected school, students were grouped by grade, and at least two classes per grade were randomly sampled. All students in the selected classes were invited to participate, with a minimum of 80 students per grade. To avoid duplicate enrollment, each participant was assigned a unique identification code. Across the three survey waves, a large, population-based sample was included, with 86,423 participants in 2022, 87,515 in 2023, and 87,557 in 2024.

### 2.2. PA and Dietary Behaviors Assessment

PA and dietary behaviors were assessed using the standardized CCDRFSS questionnaire, which has demonstrated acceptable reliability (Cronbach’s α = 0.84) and validity (RMSEA = 0.07). Detailed questionnaire items are provided in the Supplementary Materials (Table S1). PA was assessed based on self-reported frequency of moderate-to-vigorous physical activity (MVPA). Adolescents reported the number of days in the past 7 days in which they achieved at least 60 min of MVPA. Consistent with international guidelines, adolescents who achieved ≥60 min of MVPA on all 7 days were classified as meeting the PA guideline (adequate), whereas those who did not were classified as inadequate [21]. Three PA indicators were constructed: weekly PA frequency, weekday PA frequency and weekend PA frequency. Weekly PA frequency was directly derived from responses to the 7-day MVPA question. Weekend PA frequency was assessed using a specific item on MVPA participation during weekends and categorized into three levels: high (“always” or “most of the time”), moderate (“1 day”) and low (“rarely” or “0 day”). Based on the distribution of weekly and weekend PA, weekday PA frequency was further categorized into low, moderate and high levels to capture differences in the temporal distribution of PA across weekdays and weekends.

Dietary behaviors were assessed based on the frequency and variety of specific food consumption. Intake frequency was categorized as <1 time/day, 1 time/day or ≥2 times/day and intake variety was categorized as <1 type/day, 1 type/day or ≥2 types/day. Guided by the Planetary Health Diet Index framework [22,23], a plant-based Planetary Health Diet Index, PHDI-green score, was constructed to capture the core plant-based components of the diet available in the dataset. Adolescents were considered adherent to the PHDI-green pattern if they met both frequency and variety criteria for fruit and vegetable intake, defined as consuming fresh fruits and vegetables at least once per day and consuming at least one type of fresh fruit and one type of vegetable per day [24].

### 2.3. Health Outcomes Assessment

Anthropometric measurements were collected by trained personnel following standardized protocols. Height was recorded to the nearest 0.1 cm using a calibrated stadiometer, while body weight was measured to the nearest 0.1 kg with a digital scale. Body mass index (BMI) was computed as weight (kg) divided by height squared (m^2^).

Obesity classification was based on the 2007 World Health Organization (WHO) growth reference for school-aged populations [25]. Age- and sex-specific BMI z-scores were derived, with values ≥ 2 indicating obesity and values < 2 considered non-obese. BMI z-scores were calculated using the zanthro module in STATA (version 14.0; StataCorp LLC, College Station, TX, USA).

Depressive symptoms were evaluated using the Center for Epidemiologic Studies Depression Scale (CES-D), a validated 20-item self-report measure with total scores ranging from 0 to 60. The CES-D has demonstrated acceptable reliability (Cronbach’s α = 0.84) and construct validity (RMSEA = 0.07). A cutoff score of ≥16 was applied to define the presence of depressive symptoms [26].

Co-occurrence was defined as the coexistence of obesity and depressive symptoms.

### 2.4. Covariates

Age was derived from the difference between the date of birth and the date of the physical examination. Sex was obtained from official identification documents and categorized as boy or girl. Residential location was categorized as urban or rural based on administrative designation. Parental co-residence was assessed using a self-reported item asking whether the adolescent had lived with at least one parent during the past six months. Participants were categorized as living with at least one parent or living without either parent.

Data collection and quality control were conducted by trained physicians and school health professionals organized by the Zhejiang Provincial Center for Disease Control and Prevention. All investigators received standardized training and certification prior to data collection. Uniform measurement protocols and calibrated instruments were used. Daily on-site rechecks were conducted in 5% of participants, and quality control error rate was maintained below 5%, ensuring high data quality.

### 2.5. Statistical Analysis

Continuous variables were examined for normality using the Shapiro–Wilk test and are expressed as mean ± standard deviation (SD), while categorical variables are described as frequencies and proportions. Differences in demographic characteristics between boys and girls were assessed using chi-square tests. Trends in the prevalence of obesity, depressive symptoms, and their co-occurrence from 2022 to 2024 were assessed with the chi-square test for trend.

Sex-specific logistic regression analyses were conducted to explore the relationships of PA characteristics and dietary patterns with obesity, depressive symptoms, and their co-occurrence, with effect estimates presented as odds ratios (ORs) and 95% confidence intervals (CIs). To formally assess sex differences in regression coefficients while accounting for correlations across multiple health outcomes, seemingly unrelated estimation was employed, and cross-equation Wald tests were used using the suest procedure in STATA (version 14.0; StataCorp LLC, College Station, TX, USA).

Restricted cubic spline (RCS) models within the logistic regression framework were used to examine potential non-linear dose–response relationships between weekly PA, the PHDI-green score, and the model-predicted probabilities of obesity, depressive symptoms, and their co-occurrence.

Population attributable fraction (PAF) analyses were conducted separately for boys and girls to quantify the contribution of each exposure to the burden of health outcomes. As logistic regression models yield ORs, the adjusted ORs were first converted to approximate relative risks (RRs) using the formula RR = OR/[(1 − P_0_) + (P_0_ × OR)], where P_0_ denotes the prevalence of the outcome in the unexposed population [27]. This approach mitigates potential overestimation when the outcome is not rare (>10%). PAFs were then calculated for inadequate weekly PA, low-to-moderate weekend PA, low-to-moderate weekday PA, and non-adherence to PHDI-green using the standard formula PAF = Pex × (RR − 1)/[1 + Pex × (RR − 1)], where Pex denotes the prevalence of the specific exposure in the study population. PAF values range from 0% to 100%, with larger values indicating greater potential reduction in population-level disease burden.

Joint associations of PA and PHDI-green adherence were evaluated by adding an interaction term (PA × PHDI-green) to regression models. Main results are presented as ORs, with RRs used only for PAF estimation after conversion. All statistical analyses were conducted using Stata software (version 14.0; StataCorp LLC, College Station, TX, USA) and R software (version 4.2.3; R Core Team, Vienna, Austria). Statistical significance was defined as two-sided *p*-values < 0.05.

## 3. Results

### 3.1. Basic Information of Adolescents

A total of 261,495 adolescents aged 11–18 years were included in the analysis, comprising 135,309 boys (51.7%) and 126,186 girls (48.3%), with a mean age of 15.0 ± 1.7 years, as presented in Table 1. Significant sex differences were observed in obesity, depressive symptoms, their co-occurrence and indicators related to PA and dietary behaviors (all *p* < 0.001), whereas no significant regional differences were detected (*p* = 0.121). The prevalence of obesity, depressive symptoms and their co-occurrence was 6.8%, 19.5% and 1.2%, respectively.

### 3.2. Temporal Trends and Sex Disparity in Health Outcomes and Lifestyle Behaviors

Trend analyses indicated a significant increase in the prevalence of obesity from 7.14% in 2022 to 7.48% in 2024 (*p* for trend = 0.013). Similarly, the prevalence of depressive symptoms showed a statistically significant upward trend over the same period from 23.6% to 24.5% (*p* for trend = 0.003). The prevalence of co-occurrence changed from 1.2% to 1.3% with no significant trend (*p* for trend = 0.058).

**Figure 1:**
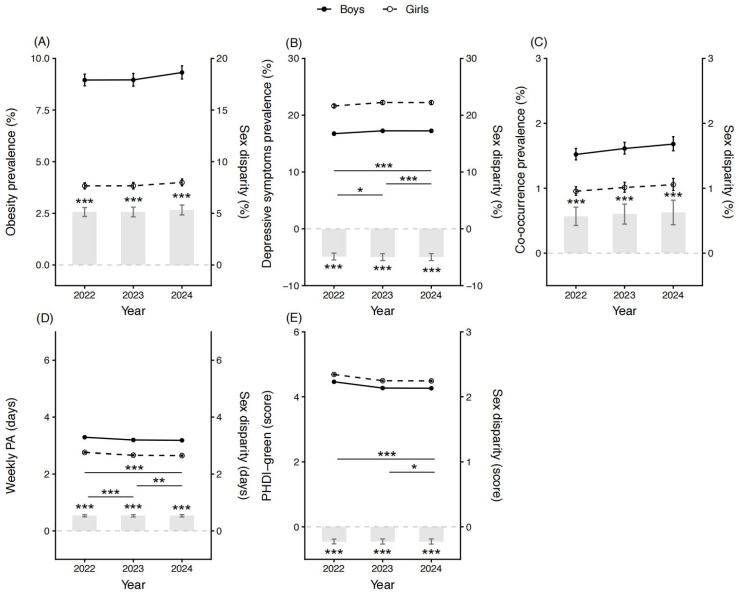
Sex disparities in health outcomes and lifestyle behaviors among adolescents, 2022–2024. Note: PA, physical activity; PHDI-green, plant-based Planetary Health Diet Index. * *p* < 0.05, ** *p* < 0.01, *** *p* < 0.001. (**A**–**E**) were adjusted for age, parental co-residence and residence.

As shown in Figure 1, after adjustment for basic sociodemographic characteristics, significant sex differences were observed. Boys consistently exhibited higher prevalences of obesity and co-occurrence than girls (all *p* < 0.001), with the magnitude of these sex differences remaining relatively stable over time. In contrast, the prevalence of depressive symptoms was significantly higher among girls than boys, with the absolute sex difference widening steadily over time (*p*_2022–2023_ = 0.016, *p*_2023–2024_ < 0.001, *p*_2022–2024_ < 0.001). Regarding lifestyle behaviors, girls consistently reported lower levels of weekly PA but higher adherence to the PHDI-green compared with boys (all *p* < 0.001), and the sex disparities in both behaviors showed a progressive widening across the study period.

### 3.3. Sex-Specific Associations of PA and PHDI-Green with Obesity, Depressive Symptoms and Their Co-Occurrence

**Figure 2:**
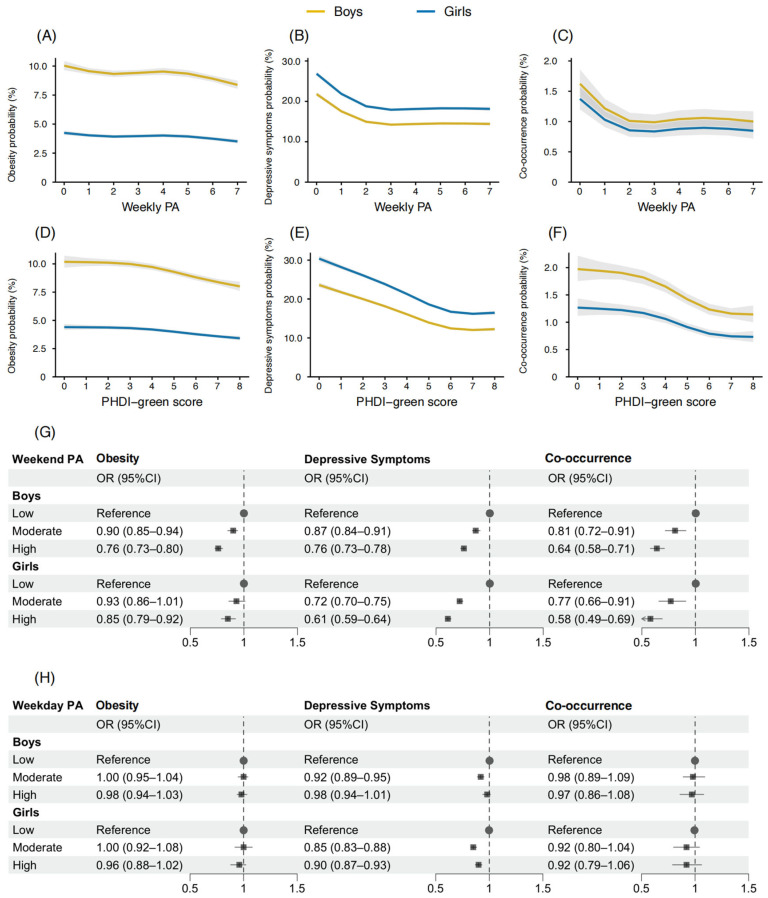
Sex-specific associations of PA and the PHDI-green score with health outcomes. Note: PA, physical activity; PHDI-green, plant-based Planetary Health Diet Index. (**A**) was adjusted for year, PHDI-green score, parental co-residence, residence, age and CES-D score. (**B**) was adjusted for year, PHDI-green score, parental co-residence, residence, age and BMI. (**C**) was adjusted for year, PHDI-green score, parental co-residence, residence and age. (**D**) was adjusted for year, weekly PA, parental co-residence, residence, age and CES-D score. (**E**) was adjusted for year, weekly PA, parental co-residence, residence, age and BMI. (**F**) was adjusted for year, weekly PA, parental co-residence, residence and age. (**G**) co-occurrence was adjusted for year, weekday PA, PHDI-green score, parental co-residence, residence and age; obesity further adjusted for CES-D score; depressive symptoms further adjusted for BMI. (**H**) co-occurrence was adjusted for year, weekend PA, PHDI-green score, parental co-residence, residence and age; obesity further adjusted for CES-D score; depressive symptoms further adjusted for BMI. Shaded areas (**A**–**F**) represent 95% confidence intervals (CIs). In panels (**G**–**H**), grey squares indicate odds ratios (ORs), horizontal lines represent 95% CIs, and grey dots denote the reference group (OR = 1). Arrows indicate that the lower bound of the 95% CI extends beyond the plotted axis range.

RCS analyses based on logistic regression models showed that, while holding covariates constant, model-predicted probabilities of obesity decreased from 4.18% (95% CI: 3.98–4.38%) to 3.48% (95% CI: 3.28–3.68%) in girls, and from 10.02% (95% CI: 9.72–10.32%) to 8.38% (95% CI: 8.08–8.68%) in boys across increasing levels of weekly PA. Similar decreasing trends were observed for depressive symptoms and co-occurrence. Specifically, the model-predicted probability of depressive symptoms decreased from 26.85% (95% CI: 26.35–27.35%) to 17.95% (95% CI: 17.65–18.25%) in girls, and from 21.48% (95% CI: 20.98–21.98%) to 14.02% (95% CI: 13.72–14.32%) in boys. For co-occurrence, the model-predicted probability decreased from 1.62% (95% CI: 1.47–1.77%) to 1.01% (95% CI: 0.86–1.16%) in boys, and from 1.38% (95% CI: 1.23–1.53%) to 0.85% (95% CI: 0.71–0.99%) in girls (Figure 2A–F).

For obesity, compared with low weekend PA and not adhering to PHDI-green, high weekend PA and adherence to PHDI-green were all associated with significantly lower odds of obesity among both boys and girls (all *p* < 0.001).

For depressive symptoms, both moderate and high weekend PA were associated with lower odds compared with low weekend PA (all *p* < 0.001), with significantly stronger protective associations in girls than in boys (all *p* for sex difference < 0.001). In contrast, adherence to the PHDI-green pattern was associated with lower odds of depressive symptoms compared with non-adherence, with a stronger inverse association observed in boys (boys: OR = 0.63, 95% CI: 0.61–0.65, *p* < 0.001; girls: OR = 0.66, 95%CI: 0.64–0.68, *p* < 0.001; *p* for sex difference < 0.001).

Regarding co-occurrence, moderate and high weekend PA, and adherence to the PHDI-green pattern were each associated with lower odds of co-occurrence in both boys and girls (all *p* < 0.001), with no significant sex differences observed. Specifically, moderate weekend PA was associated with reduced odds (boys: OR: 0.81, 95% CI: 0.72–0.91; girls: OR: 0.77, 95% CI: 0.66–0.91), as was high weekend PA (boys: OR: 0.64, 95% CI: 0.58–0.71; girls: OR: 0.58, 95% CI: 0.49–0.69). Similarly, adherence to the PHDI-green pattern was associated with lower odds of co-occurrence (boys: OR = 0.66, 95% CI: 0.60–0.73; girls: OR = 0.72, 95% CI: 0.64–0.81; Figure 2G,H and Table S2).

**Figure 3:**
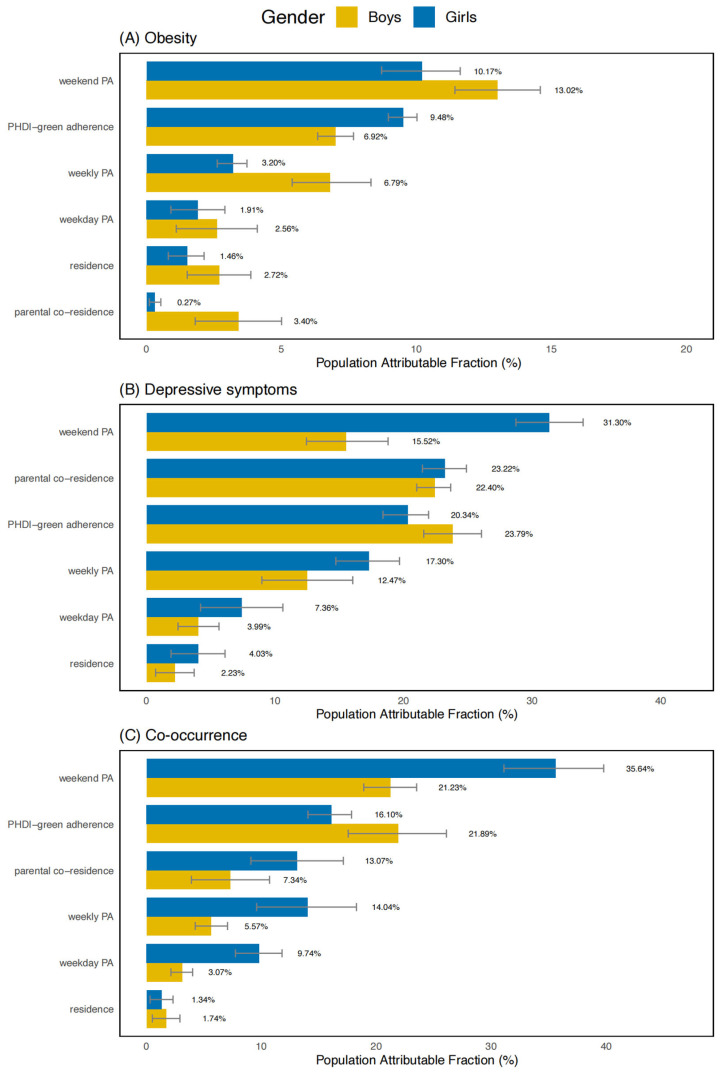
Sex-specific population attributable fraction of PA and the PHDI-green adherence for health outcomes. Note: PA, physical activity; PHDI-green, plant-based Planetary Health Diet Index. (**A**) was adjusted for year, age and CES-D score. (**B**) was adjusted for year, age and BMI. (**C**) was adjusted for year and age.

PAF analyses based on RRs suggested that low weekend PA accounted for the largest proportions of cases for obesity (10.17%, 95% CI: 9.35–10.99%), depressive symptoms (31.30%, 95% CI: 29.45–33.15%), and their co-occurrence (35.64%, 95% CI: 33.80–37.48%) among girls. In boys, non-adherence to the PHDI-green pattern accounted for higher proportions of cases for obesity (6.92%, 95% CI: 6.05–7.79%), depressive symptoms (23.79%, 95% CI: 22.25–25.33%), and co-occurrence (21.89%, 95% CI: 20.40–23.38%). These findings are also illustrated in Figure 3.

**Figure 4:**
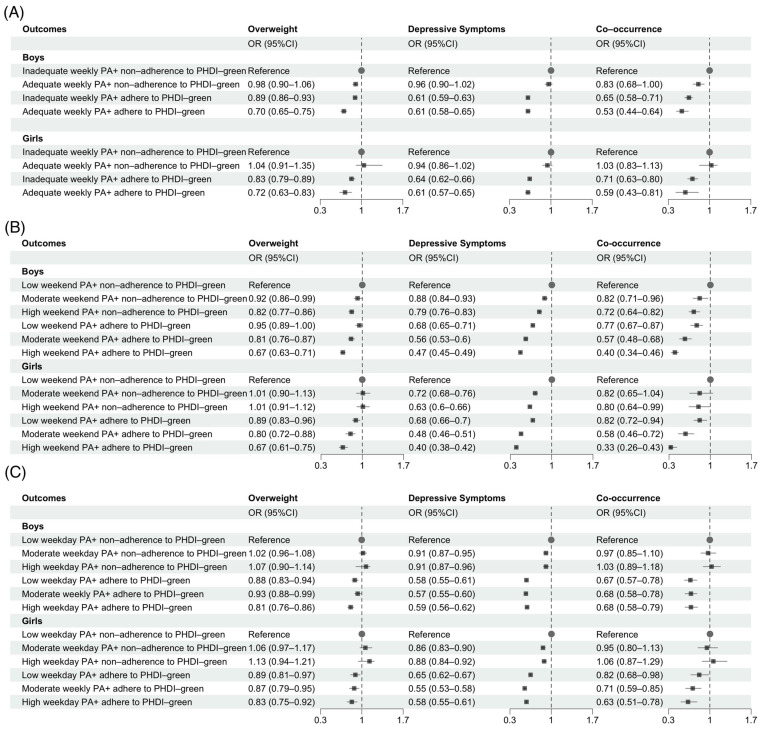
Joint associations of PA and PHDI-green adherence with obesity, depressive symptoms and their co-occurrence. Note: PA, physical activity; PHDI-green, plant-based Planetary Health Diet Index. (**A**) Co-occurrence was adjusted for year, parental co-residence, residence and age; obesity further adjusted for CES-D score; depressive symptoms further adjusted for BMI. (**B**) Co-occurrence was adjusted for year, weekday PA, parental co-residence, residence and age; obesity further adjusted for CES-D score; depressive symptoms further adjusted for BMI. (**C**) Co-occurrence was adjusted for year, weekend PA, parental co-residence, residence and age; obesity further adjusted for CES-D score; depressive symptoms further adjusted for BMI. Grey squares indicate odds ratios (ORs), horizontal lines represent 95% confidence intervals (CIs), and grey dots denote the reference group (OR = 1). Arrows indicate that the lower bound of the 95% CI extends beyond the plotted axis range.

In the joint analysis of PA and PHDI-green adherence, compared with inadequate weekly PA and non-adherence to PHDI-green, adolescents with adequate weekly PA and adherence to PHDI-green showed the lowest odds of obesity, depressive symptoms and their co-occurrence in both boys and girls (Figure 4A). High weekend PA combined with PHDI-green adherence was further associated with substantially reduced odds of co-occurrence (boys: OR = 0.40, 95% CI: 0.34–0.46; girls: OR = 0.33, 95% CI: 0.26–0.43; Figure 4B). Sex-specific patterns were also observed for weekday PA. Low weekday PA with adherence to PHDI-green showed the strongest inverse association with co-occurrence in boys (OR = 0.67, 95% CI: 0.57–0.78), whereas among girls, high weekday PA with PHDI-green adherence showed the strongest association (OR = 0.63, 95% CI: 0.51–0.78; Figure 4C).

## 4. Discussion

In this study, both PA and adherence to the PHDI-green dietary pattern were robustly associated with obesity, depressive symptoms and their co-occurrence. Clear sex disparities were observed: adherence to PHDI-green showed stronger associations among boys, whereas PA was more strongly associated with lower odds of these outcomes among girls. Notably, weekend PA demonstrated stronger associations with favorable health outcomes than weekday PA across adolescents. Together, these findings suggest the importance of considering sex-specific and time-specific lifestyle behaviors in relation to adolescent health.

The prevalence of obesity and depressive symptoms among adolescents in Zhejiang Province showed a consistent upward trend between 2022 and 2024, with co-occurrence becoming increasingly prominent. Specifically, the prevalence of obesity among boys was 9.4%, which is comparable to the global prevalence reported for boys [1]. The prevalence of depressive symptoms reached 19.5%, reflecting an increase of 4.7% compared with estimates from 2018 to 2021 [28]. Consistent with these observations, projections from the Global Burden of Disease study further indicate a continuous upward trend in the future burden of depressive disorders among adolescents [29]. Therefore, these findings underscore the growing public health relevance of integrated prevention and control strategies targeting both physical and mental health in adolescents.

Previous studies have primarily focused on total PA in relation to physical and mental health outcomes [21,30,31]. Building on this evidence, the present study suggests that the temporal patterning of PA may also be relevant, with weekend PA showing consistent associations with lower odds of obesity, depressive symptoms, and their co-occurrence. These associations persisted after adjustment for dietary behaviors. Similar patterns have been reported in adult “weekend warrior” populations [15], supporting the possibility that the accumulation of PA within a shorter time frame may be differentially associated with health outcomes. From a biological perspective, adolescents typically experience delayed circadian rhythms, such that mandatory early rising on weekdays leads to misalignment with physiological peaks in body temperature and cortisol secretion. In contrast, weekend PA is more likely to occur after natural awakening during daylight hours, thereby facilitating better alignment with endogenous circadian rhythms [32]. In addition, weekend exercise more often involves participation with family members, which may enhance enjoyment and social connectedness, potentially through dopaminergic and oxytocin-related pathways, thus amplifying anti-depressive effects [33]. Supporting this interpretation, students living in single-parent households tend to engage in less weekend PA [34], further demonstrating that the health advantages of weekend PA over weekday PA may be closely linked to family engagement and parent–child interactions [35].

Previous studies have linked dietary habits to obesity and depressive symptoms, but most have focused on individual foods, food processing, or unhealthy eating patterns [36,37,38]. Research examining adherence to the PHDI, which promotes primarily plant-based diets for health and environmental benefits, in relation to adolescent outcomes remains scarce. For example, cohort evidence suggests that higher adherence to plant-focused diets is associated with lower odds of experiencing adverse depressive symptom trajectories, though this finding has mainly been observed in older adults [39]. Likewise, a recent study assessing the PHDI for weight management in children highlighted its role in supporting healthy weight but did not investigate links with depressive symptoms or their co-occurrence in adolescents [40].

Focusing on vegetable and fruit intake, the present study found that greater adherence to the PHDI-green pattern was linked to reduced odds of depressive symptoms in adolescents. This protective association may involve several interconnected biological mechanisms. Diets rich in vegetables and fruits provide abundant antioxidants, such as beta-carotene and vitamins C and E, which can help mitigate oxidative stress and neuroinflammation involved in depression [41]. Moreover, higher fiber intake may support a more diverse gut microbial community and increase short-chain fatty acid production, which may influence brain function through gut–brain signaling, potentially improving mood regulation [42]. Systematic reviews have further indicated that higher intake of plant foods is associated with lower depressive symptoms in adolescents, possibly via enhanced serotonin synthesis from folate and other micronutrients, along with decreased systemic inflammation [43]. These mechanisms underscore the modifiable nature of dietary interventions in adolescent mental health, warranting further longitudinal studies to confirm causality.

This study revealed significant sex disparities in the prevalence of health outcomes and lifestyle behaviors, as well as in their associations. Boys showed higher rates of obesity and co-occurrence, consistent with international data showing that boys generally exhibit higher obesity prevalence in many economically developed and moderately developed countries [44]. In contrast, girls showed higher rates of depressive symptoms, with these disparities widening over time. This observation aligns with longitudinal evidence showing that girls experience steeper increases in depressive symptoms from adolescence to early adulthood than boys [45]. Such sex differences in health outcomes may reflect interactions between pubertal development and lifestyle behaviors [46,47]. Notably, the widening gap in depressive symptoms may partly reflect diverging lifestyle patterns: girls showed a progressively larger decline in physical activity, particularly on weekends, while boys maintained lower adherence to the PHDI-green. Given the stronger protective association of physical activity with mental health outcomes in girls, these sex-specific behavioral trends likely contribute to the increasing female disadvantage in depressive symptoms over the study period. This temporal correspondence between behavioral divergence and health disparities underscores the need for early, sex-responsive preventive strategies in adolescence.

This study further revealed that girls engaged in substantially lower levels of PA than boys, whereas boys showed poorer adherence to the PHDI-green, with both gaps widening over time. These sex-specific differences in PA and diet may be related to differences in health outcomes [48,49]. Weekend PA showed the strongest associations with lower odds of co-occurrence among girls, whereas adherence to the PHDI-green pattern showed stronger associations among boys. These differential associations may reflect sex-specific metabolic and psychosocial factors. During puberty, girls experience transient insulin resistance [50] and heightened stress reactivity [51], which may partly explain why weekend PA showed stronger associations with these outcomes in girls.

In boys, diets emphasizing plant foods and high in fiber and phytonutrients may more efficiently influence systemic inflammation and gut–brain signaling, thereby affecting both metabolic and mental health [52,53]. Beyond their physiological benefits, plant-based dietary patterns are also increasingly recognized for their environmental sustainability [54]. Current evidence suggests that such diets contribute to reduced greenhouse gas production, lower land demand, and minimized biodiversity loss compared with animal-based diets, underscoring their combined benefits for human and environmental health [55,56,57]. This synergy between nutritional quality and ecological considerations is particularly important during adolescence, a formative stage for shaping long-term eating behaviors [58].

Specifically, higher dietary fiber intake supports the generation of short-chain fatty acids, including butyrate, through microbial fermentation in the gut, which can suppress pro-inflammatory cytokines, strengthen intestinal barrier function, and improve metabolic regulation by enhancing insulin sensitivity and maintaining energy balance [59]. Reduced intestinal permeability may further limit the translocation of endotoxins such as lipopolysaccharide, thereby attenuating chronic low-grade systemic inflammation [60]. In parallel, metabolites derived from gut microbiota can affect brain function through neural, hormonal, and immune signaling pathways, including regulation of the hypothalamic–pituitary–adrenal axis and neurotransmitter networks, which are closely involved in mood regulation and depressive symptoms [61,62,63]. Emerging evidence further suggests that sex differences in immune function and gut microbiota composition may contribute to sex-specific responses to dietary exposures, as distinct immune profiles and microbial communities can differentially influence inflammatory responses, metabolic processes, and gut–brain axis signaling [64,65]. Psychosocial factors may further amplify these sex-specific effects, as these factors are known to interact with both immune regulation and gut–brain axis function [66,67].

Weekend PA often involves social interaction and green space exposure [68], which may provide stronger buffering against the academic and social stressors that disproportionately affect girls’ mental health, whereas higher dietary quality appears to offer relatively greater benefits for boys. Notably, the progressive polarization of lifestyle behaviors by sex during adolescence likely exacerbates underlying biological vulnerabilities and psychosocial stressors, thereby accelerating inequalities in obesity, depressive symptoms, and their co-occurrence if unaddressed. From a public health perspective, these findings highlight a critical window for sex-tailored interventions that prioritize increased PA among girls and improved PHDI-green adherence among boys, thereby mitigating emerging sex-based health disparities. Importantly, promoting adherence to plant-forward dietary patterns may yield co-benefits by simultaneously improving adolescent health outcomes and reducing the environmental footprint of food systems, thereby supporting sustainable public health strategies.

This study has several strengths. It analyzed a large, population-based cohort of adolescents, improving the reliability of the findings. By considering the timing of physical activity, our results emphasize the importance of weekend activity for adolescent health. Additionally, employing the PHDI-green enabled us to examine associations between fruit and vegetable consumption, obesity, depressive symptoms, and their co-occurrence, while analyses stratified by sex highlighted potential sex-specific prevention priorities. Some limitations should be noted. First, this research is a secondary analysis of school-based health examination data; despite the large sample, selection bias cannot be fully excluded. Second, the observational design prevents establishing causality, and observed links between PA, diet, and health outcomes should be interpreted cautiously. Third, physical activity, dietary intake, and depressive symptoms were self-reported, introducing possible recall or social desirability bias. Future studies with extended follow-up and objective measurements are needed to validate these results and clarify causal relationships.

## 5. Conclusions

This large population-based study of adolescents in Zhejiang Province underscores the complementary roles of physical activity and a plant-based diet in promoting adolescent health, with notable sex-specific differences. Stronger inverse associations for weekend PA and more pronounced associations for PHDI-green adherence among boys suggest that both the timing and type of lifestyle behaviors may be important for adolescent health. Future studies should investigate the mechanisms behind these sex-specific associations, including hormonal, behavioral, or gut–brain axis pathways, and examine the long-term impact of combined lifestyle patterns on obesity, depressive symptoms, and their co-occurrence.

## Figures and Tables

**Table 1:** Characteristics of adolescents, 2022–2024 (N = 261,495).

Variables	Total (N = 261,495)	Sex Group		*p* Value
		Boys (N = 135,309)	Girls (N = 126,186)	
Height (cm)	163.9 (8.7)	167.8 (8.9)	159.7 (6.0)	<0.001
Weight (kg)	56.2 (12.8)	59.5 (14.2)	52.6 (10.0)	<0.001
BMI (kg/m^2^)	20.8 (3.7)	20.9 (4.0)	20.6 (3.4)	<0.001
Obesity (%)				
Yes	17,731 (6.8)	12,684 (9.4)	5047 (4.0)	<0.001
No	243,764 (93.2)	122,625 (90.6)	121,139 (96.0)	
Depressive symptoms (%)				
Yes	50,914 (19.5)	23,114 (17.1)	27,800 (22.0)	<0.001
No	210,581 (80.5)	112,195 (82.9)	98,386 (78.0)	
Co-occurrence (%)				
Yes	3262 (1.2)	2056 (1.5)	1206 (1.0)	<0.001
No	258,233 (98.8)	133,253 (98.5)	124,980 (99.0)	
Weekly PA (%)				
Inadequate	232,560 (88.9)	115,682 (85.5)	116,878 (92.6)	<0.001
Adequate	28,935 (11.1)	19,627 (14.5)	9308 (7.4)	
Weekend PA (%)				
Low	134,854 (51.6)	56,409 (41.7)	78,445 (62.2)	<0.001
Moderate	47,923 (18.3)	26,176 (19.3)	21,747 (17.2)	
High	78,718 (30.1)	52,724 (39.0)	25,994 (20.6)	
Weekday PA (%)				
Low	101,330 (38.8)	50,177 (37.1)	51,153 (40.5)	<0.001
Moderate	91,863 (35.1)	46,921 (34.7)	44,942 (35.6)	
High	68,302 (26.1)	38,211 (28.2)	30,091 (23.8)	
Daily fruit variety (%)				
<1 type/day	64,353 (24.6)	36,171 (26.7)	28,182 (22.3)	<0.001
1 type/day	116,196 (44.4)	59,234 (43.8)	56,962 (45.1)	
≥2 types/day	80,946 (31.0)	39,904 (29.5)	41,042 (32.5)	
Daily vegetable variety (%)				
<1 type/day	20,436 (7.8)	11,394 (8.4)	9042 (7.2)	<0.001
1 type/day	79,314 (30.3)	41,731 (30.8)	37,583 (29.8)	
≥2 types/day	161,745 (61.9)	82,184 (60.7)	79,561 (63.1)	
Daily fruit frequency (%)				
<1 time/day	112,754 (43.1)	61,629 (45.5)	51,125 (40.5)	<0.001
1 time/day	114,669 (43.9)	56,032 (41.4)	58,637 (46.5)	
≥2 times/day	34,072 (13.0)	17,648 (13.0)	16,424 (13.0)	
Daily vegetable frequency (%)				
<1 time/day	52,652 (20.1)	29,070 (21.5)	23,582 (18.7)	<0.001
1 time/day	98,693 (37.7)	50,653 (37.4)	48,040 (38.1)	
≥2 times/day	110,150 (42.1)	55,586 (41.1)	54,564 (43.2)	
PHDI-green adherence (%)				
Yes	129,368 (49.5)	63,504 (46.9)	65,864 (52.2)	<0.001
No	132,127 (50.5)	71,805 (53.1)	60,322 (47.8)	
Parental co-residence (%)				
Yes	129,368 (49.5)	63,504 (46.9)	65,864 (52.2)	<0.001
No	132,127 (50.5)	71,805 (53.1)	60,322 (47.8)	
Residence (%)				
rural	122,037 (46.7)	63,082 (46.6)	8955 (46.7)	0.611
urban	139,458 (53.3)	72,227 (53.4)	67,231 (53.3)	

Note: PA, physical activity; PHDI-green, plant-based Planetary Health Diet Index. Continuous variables (height, weight, BMI) are expressed as mean ± SD; categorical variables (obesity, depressive symptoms, co-occurrence, weekly PA, weekend PA, weekday PA, daily fruit variety, daily vegetable variety, daily fruit frequency, daily vegetable frequency, PHDI-green adherence, parental co-residence, residence) are expressed as N (%).

## Data Availability

The data are not publicly available due to privacy. The raw data supporting the conclusions of this article will be made available upon request.

## Supplementary Materials

The following supporting information can be downloaded at https://www.mdpi.com/article/10.3390/nu18081232/s1. Table S1: Physical Activity and Dietary Behavior Questionnaire Items; Table S2: Associations of PA and PHDI-Green Adherence with the Risk of Obesity, Depressive Symptoms and their Co-occurrence.
