# Supplementary material for: Sex Differences in the Associations of Physical Activity and Planetary Health Diet with Obesity and Depressive Symptoms Among Adolescents in Zhejiang Province: An Observational Study
Source: Nutrients. 2026 Apr 14;18(8):1232. doi: 10.3390/nu18081232 (PMC13118374; doi:10.3390/nu18081232)
Supplement: Supplementary file 1 [file nutrients-18-01232-s001.zip › nutrients-4208789-supplementary.pdf]

Supplementary File

Table S1. Physical Activity and Dietary Behaviors Questionnaire Items.

Question	Option	Standard
**A. physical activity**		
1. During the past seven days, on how many days did you engage in at least 60 minutes of moderate-to-vigorous physical activity per day (cumulative allowed)?<br>(Moderate-to-vigorous physical activity refers to activities that cause rapid breathing or increased heart rate, such as running, basketball, football, swimming, jumping rope, or carrying heavy objects.)	0 day 1 day<br>2 days 3 days<br>4 days 5 days<br>6 days 7 days	Weekend physical activity (PA) was categorized into three levels: high (“always” or “most of the time”), moderate (“1 day”) and low (“rarely” or “0 day”) For weekday PA assessment, weekend PA was first converted into numeric values, with high weekend PA assigned as 2 days and low weekend PA assigned as 0 days. Weekday PA was then calculated by subtracting weekend PA days from weekly PA (range: 0–7 days), yielding weekday PA ranging from 0 to 5 days. Weekday PA was categorized into three levels: high (4-5 day), moderate (2-3 day) and low (0-1 day).
2. During the past seven days, on weekends or public holidays, how often did you engage in at least 60 minutes of moderate-to-vigorous physical activity per day (cumulative allowed)?	always<br>most of the time<br>1 day<br>rarely<br>0 day	
**B. dietary behaviors**		
3. How many times per day do you usually consume fresh fruit (excluding canned fruit)?	<1 time/day<br>1 time/day<br>≥2 times/day	The PHDI-green score (range: 0-8) was calculated by summing responses from the four dietary items related to fruit and vegetable intake (Questions 3-6). Each item was scored as follows:<br>Frequency items (times/day):<br>< 1 time/day = 0 points<br>1 time/day = 1 point<br>≥ 2 times/day = 2 points<br>Variety items (types/day):<br>< 1 type/day = 0 points<br>1 type/day = 1 point<br>≥ 2 types/day = 2 points<br>Higher PHDI-green scores indicate greater adherence to a plant-based dietary pattern characterized by higher frequency and variety of fruit and vegetable consumption.
4. How many types of fresh fruit do you usually consume per day?	<1 type/day<br>1 type/day<br>≥2 types/day	
5. How many times per day do you usually consume vegetables (raw or cooked, such as salads, raw vegetables, or cooked vegetables)?	<1 time/day<br>1 time/day<br>≥2 times/day	
6. How many types of vegetables do you usually consume per day?	<1 type/day<br>1 type/day<br>≥2 types/day	

Table S2. Associations of PA and PHDI-Green Adherence with the Risk of Obesity, Depressive Symptoms and their Co-occurrence.

**Outcomes**	**Variables**	**Adjusted covariate**	**Boys**		**Girls**		***p for sex difference***
			***OR* (95% *CI*)**	***p* value**	***OR* (95% *CI*)**	***p* value**	
Obesity	Weekly PA						
	Frequency	Model 1	0.98 (0.97–0.99)	<0.001	1.01 (0.99–1.02)	0.326	<0.001
		Model 2	0.98 (0.97–1.00)	<0.001	0.98 (0.96–1.01)	<0.001	0.931
	Weekend PA						
	Low		Ref	Ref	Ref	Ref	
	Moderate	Model 1	0.91 (0.86–0.95)	<0.001	0.99 (0.92–1.07)	<0.001	0.058
		Model 3	0.90 (0.85–0.94)	<0.001	0.93 (0.86–1.01)	0.085	0.399
	High	Model 1	0.78 (0.75–0.81)	<0.001	0.93 (0.86–1.00)	0.049	<0.001
		Model 3	0.76 (0.73–0.80)	<0.001	0.85 (0.79–0.92)	<0.001	0.144
	Weekday PA						
	Low		Ref	Ref	Ref	Ref	
	Moderate	Model 1	1.02 (0.98–1.06)	0.383	1.03 (0.96–1.10)	0.426	0.854
		Model 4	1.00 (0.95–1.04)	0.166	1.00 (0.92–1.08)	0.23	0.885
	High	Model 1	1.00 (0.96–1.05)	0.467	1.08 (0.98–1.12)	0.067	0.017
		Model 4	0.98 (0.94–1.03)	0.417	0.96 (0.88–1.02)	0.235	0.447
	PHDI-green adherence						
	No		Ref	Ref	Ref	Ref	
	Yes	Model 1	0.92 (0.89–0.96)	<0.001	0.88 (0.83–0.93)	<0.001	<0.001
		Model 5	0.87 (0.84–0.90)	<0.001	0.82 (0.77–0.87)	<0.001	0.089
Depressive Symptoms	Weekly PA						
	Frequency	Model 1	0.93 (0.93–0.94)	<0.001	0.91 (0.90–0.91)	<0.001	<0.001
		Model 6	0.96 (0.95–0.97)	<0.001	0.94 (0.93–0.95)	<0.001	<0.001
	Weekend PA						
	Low		Ref	Ref	Ref	Ref	
	Moderate	Model 1	0.79 (0.76–0.82)	<0.001	0.63 (0.60–0.65)	<0.001	<0.001
		Model 7	0.87 (0.84–0.91)	<0.001	0.72 (0.70–0.75)	<0.001	<0.001
	High	Model 1	0.67 (0.65–0.69)	<0.001	0.53 (0.51–0.55)	<0.001	<0.001
		Model 7	0.76 (0.73–0.78)	<0.001	0.61 (0.59–0.64)	<0.001	<0.001
	Weekday PA						
	Low		Ref	Ref	Ref	Ref	
	Moderate	Model 1	0.91 (0.88–0.94)	<0.001	0.80 (0.78–0.82)	<0.001	<0.001
		Model 8	0.92 (0.89–0.95)	<0.001	0.85 (0.83–0.88)	<0.001	<0.001
	High	Model 1	0.86 (0.83–0.89)	<0.001	0.77 (0.75–0.80)	<0.001	<0.001
		Model 8	0.98 (0.94–1.01)	0.18	0.90 (0.87–0.93)	<0.001	0.003
	PHDI-green adherence						
	No		Ref	Ref	Ref	Ref	
	Yes	Model 1	0.56 (0.54–0.58)	<0.001	0.59 (0.57–0.61)	<0.001	0.008
		Model 9	0.63 (0.61–0.65)	<0.001	0.66 (0.64–0.68)	<0.001	0.040

Table S2. Cont.

Outcomes	Variables	Adjusted covariate	Boys		Girls		*p for sex difference*
			***OR* (95% *CI*)**	***p* value**	***OR* (95% *CI*)**	***p* value**	
	Weekly PA						
	Frequency	Model 1	0.98 (0.97–0.99)	<0.001	1.01 (0.99–1.02)	0.326	<0.001
		Model 2	0.98 (0.97–1.00)	<0.001	0.98 (0.96–1.01)	<0.001	0.931
	Weekend PA						
	Low		Ref	Ref	Ref	Ref	
	Moderate	Model 1	0.91 (0.86–0.95)	<0.001	0.99 (0.92–1.07)	<0.001	0.058
		Model 3	0.90 (0.85–0.94)	<0.001	0.93 (0.86–1.01)	0.085	0.399
	High	Model 1	0.78 (0.75–0.81)	<0.001	0.93 (0.86–1.00)	0.049	<0.001
		Model 3	0.76 (0.73–0.80)	<0.001	0.85 (0.79–0.92)	<0.001	0.144
Obesity	Weekday PA						
	Low		Ref	Ref	Ref	Ref	
	Moderate	Model 1	1.02 (0.98–1.06)	0.383	1.03 (0.96–1.10)	0.426	0.854
		Model 4	1.00 (0.95–1.04)	0.166	1.00 (0.92–1.08)	0.23	0.885
	High	Model 1	1.00 (0.96–1.05)	0.467	1.08 (0.98–1.12)	0.067	0.017
		Model 4	0.98 (0.94–1.03)	0.417	0.96 (0.88–1.02)	0.235	0.447
	PHDI-green adherence						
	No		Ref	Ref	Ref	Ref	
	Yes	Model 1	0.92 (0.89–0.96)	<0.001	0.88 (0.83–0.93)	<0.001	<0.001
		Model 5	0.87 (0.84–0.90)	<0.001	0.82 (0.77–0.87)	<0.001	0.089

Note: PA, physical activity; PHDI-green, Planetary Health Diet Index fruit and vegetable score. Model 1 was adjusted for year. Model 2 was adjusted for year, PHDI-green score, parental co-residence, residence, age and CES-D score. Model 3 was adjusted for year, weekday PA, PHDI-green score, parental co-residence, residence, age and CES-D score. Model 4 was adjusted for year, weekend PA, PHDI-green score, parental co-residence, residence, age and CES-D score. Model 5 was adjusted for year, weekly PA, parental co-residence, residence, age and CES-D score. Model 6 was adjusted for year, PHDI-green score, parental co-residence, residence, age and BMI. Model 7 was adjusted for year, weekday PA, PHDI-green score, parental co-residence, residence, age and BMI. Model 8 was adjusted for year, weekend PA, PHDI-green score, parental co-residence, residence, age and BMI. Model 9 was adjusted for year, weekly PA, parental co-residence, residence, age and BMI. Model 10 was adjusted for year, PHDI-green score, parental co-residence, residence and age. Model 11 was adjusted for year, weekday PA, PHDI-green score, parental co-residence, residence and age. Model 12 was adjusted for year, weekend PA, PHDI-green score, parental co-residence, residence and age. Model 13 was adjusted for year, weekly PA, parental co-residence, residence and age.
